# Rare Acute Presentation of a Low-Grade Thymic Neuroendocrine Tumor

**DOI:** 10.7759/cureus.10850

**Published:** 2020-10-08

**Authors:** Kush Gupta, Poras Patel, Saikrishna Patibandla, Raheel Anwar, Elizabeth Guevara

**Affiliations:** 1 Internal Medicine, Kasturba Medical College, Mangalore, IND; 2 Hematology/Oncology, The Brooklyn Hospital Center, Brooklyn, USA; 3 Cardiology, West Virginia University, Morgantown, USA; 4 Internal Medicine, The Brooklyn Hospital Center, Brooklyn, USA

**Keywords:** neuroendocrine tumor, thymus, mediastinal mass, congestive heart failure, carcinoid tumor

## Abstract

Primary neuroendocrine tumor (NET) of the thymus is very rare. Here we report an unusual presentation of grade-I typical thymic NET in a 63-year-old female who presented with signs and symptoms of congestive heart failure (CHF) due to the mass effect from the huge tumor. A computed tomography (CT) scan of the chest revealed a mediastinal mass measuring 16.4 x 12 x 15.3 cm displacing most of the left lung parenchyma, with mass effect on the heart, and encasing the ascending aorta and main pulmonary trunk. Pathology report from the thymic mass biopsy showed tumor cells strongly expressing synaptophysin, chromogranin A, and cluster of differentiation (CD)56 markers. The diagnosis was consistent with grade-I typical thymic NET based on low Ki-67 and morphology. The patient was not in agreement for acute surgery or oncological treatment options. Thus, the plan was made to embolize the arteries from the right coronary artery that were feeding the mass in an effort to shrink the size with goals of future surgical resection. However, given the advanced stage of the diagnosis with mass effect on the heart and the patient’s reluctance to consider the main definitive treatment options, the prognosis was extremely poor and the patient eventually passed away.

## Introduction

The primary role of the thymus is to process and develop mature lymphocytes, which become T-lymphocytes upon release into the circulation [1]. The most common neoplasms of the thymus are thymoma and thymic carcinoma, both arising from the epithelial cells of the thymus.

Malignancies of the thymus are relatively rare (incidence 0.13 cases per 100,000 population in the United States) [1]. Among all the primary thymic malignancies, neuroendocrine neoplasms are the least common (2%-5%) with an annual incidence rate of 0.2 per million [1].

A carcinoid tumor is a specific type of neuroendocrine tumor (NET), which arises from hormone-producing cells of the body’s neuroendocrine system. German pathologist, Siegfried Oberndorger, first described carcinoid in 1907 as Karzinoide, or “carcinoma-like”, due to the unique feature of behaving like a benign tumor despite having a malignant appearance microscopically [2]. Primary NET of the thymus, also known as thymic carcinoid tumor, is an extremely rare entity, believed to have a more indolent course and aggressive features when compared to other NETs.

Based on the histological features, thymic NETs are divided into three categories as low-grade (well differentiated), intermediate-grade (moderately differentiated), and high-grade (poorly differentiated) [3]. The NETs of the thymus are classified into four types: typical carcinoid, atypical carcinoid, small cell carcinoma, and large cell neuroendocrine carcinoma [4]. They share similar histological appearance with lung carcinoids and have been considered a single group of tumors in their classification. However, several factors make them unique, such as their presentation at an advanced stage, rare incidence, poor prognosis, aggressive nature, association with endocrinopathies, and their prevalence in Caucasian and Asian men make them unique [5].

Although most thymic carcinoid tumors are found incidentally during routine imaging [6], patients may exhibit symptoms related to mass effect or invasion of mediastinal and other thoracic structures [7,8]. Here, we introduce a very unique case of primary grade-I typical thymic NET in an African American woman which remained indolent over many years, growing slowly with the help of feeding arteries arising from the coronary artery, and subsequently presenting with abrupt symptoms due to the mass effect of the tumor on the heart, lung, and surrounding vascular structures.

## Case presentation

**Figure 1:**
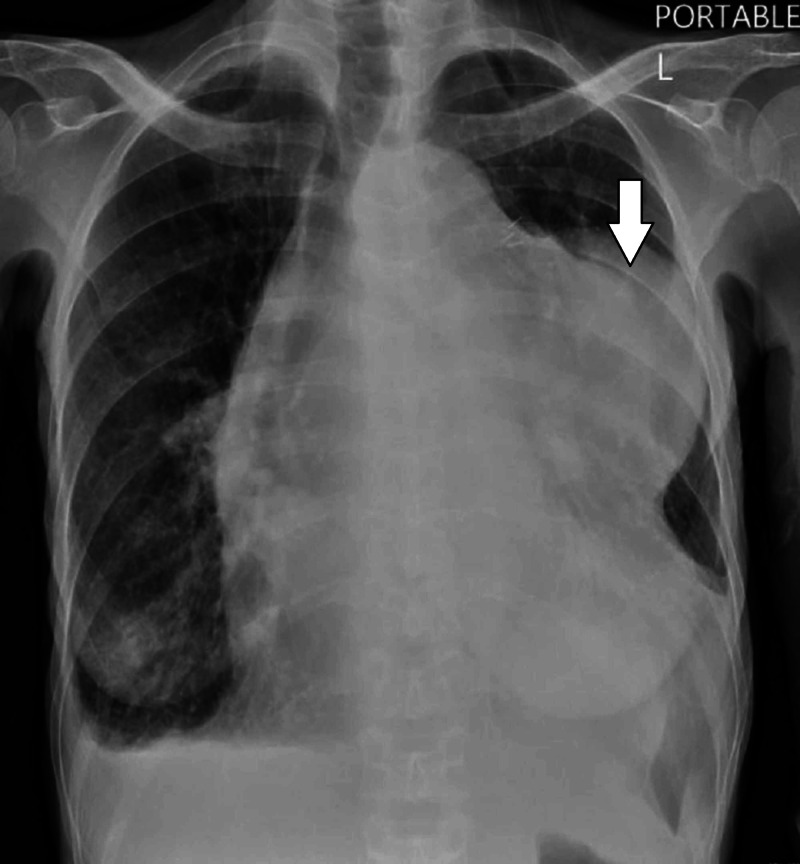
X-ray of the chest

**Figure 2:**
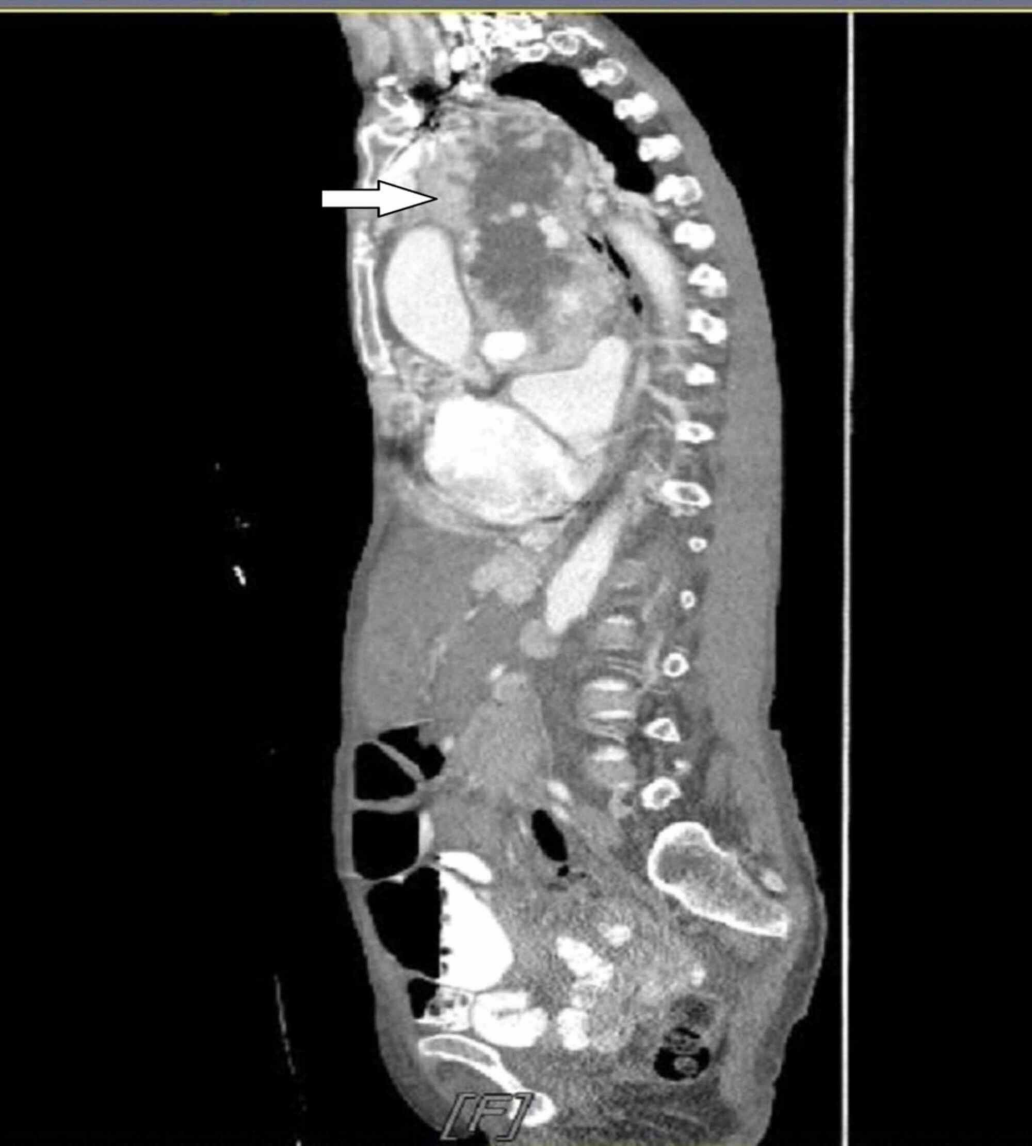
CT scan: sagittal section of the chest and abdomen revealing a large upper anterior mediastinal mass with central necrosis and with a mass effect on the heart

**Figure 3:**
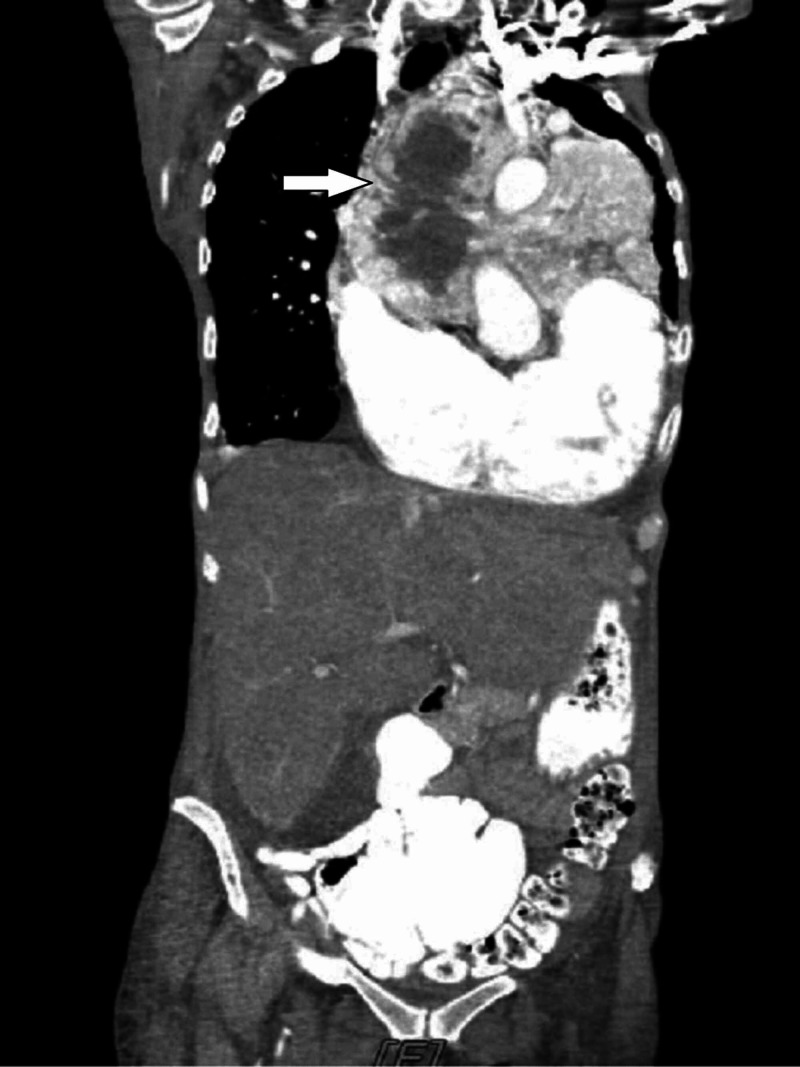
CT scan: coronal section of the chest and abdomen shows a large upper anterior mediastinal mass, displacing most of the left lung parenchyma, with mass effect on the heart

A 63-year-old African American female presented to the emergency room with progressively worsening dyspnea associated with new-onset orthopnea, bilateral lower extremity edema, and weight-loss of one-month duration. About 20 years ago, the patient was notified of an incidental finding of a mediastinal mass; however, no further intervention was done at that time, and the tumor was neglected over the years due to its indolent course. On physical examination, she was noted to have tachycardia, labored breathing, bilateral diminished breath sounds with crackles and rhonchi, and 3+ lower extremity pitting edema. Laboratory results were significant for a brain natriuretic peptide (BNP) of 2709 pg/mL, albumin 2.6 g/dL, and hemoglobin 9 g/dL. A solid mediastinal mass was found on an initial chest X-ray (Figure 1). A computed tomography (CT) scan of the chest revealed a large upper anterior mediastinal mass, measuring 16.4 x 12 x 15.3 cm displacing most of the left lung parenchyma, exerting a mass effect on the heart, and encasing the ascending aorta and main pulmonary trunk (Figures 2-3). The ejection fraction was significantly compromised to 28.7% on transthoracic echocardiogram (TTE). Coronary angiogram revealed 90% stenosis of the ostial left main coronary artery, and a large branch originating from the proximal right coronary artery (RCA) supplying the mediastinal mass.

**Figure 4:**
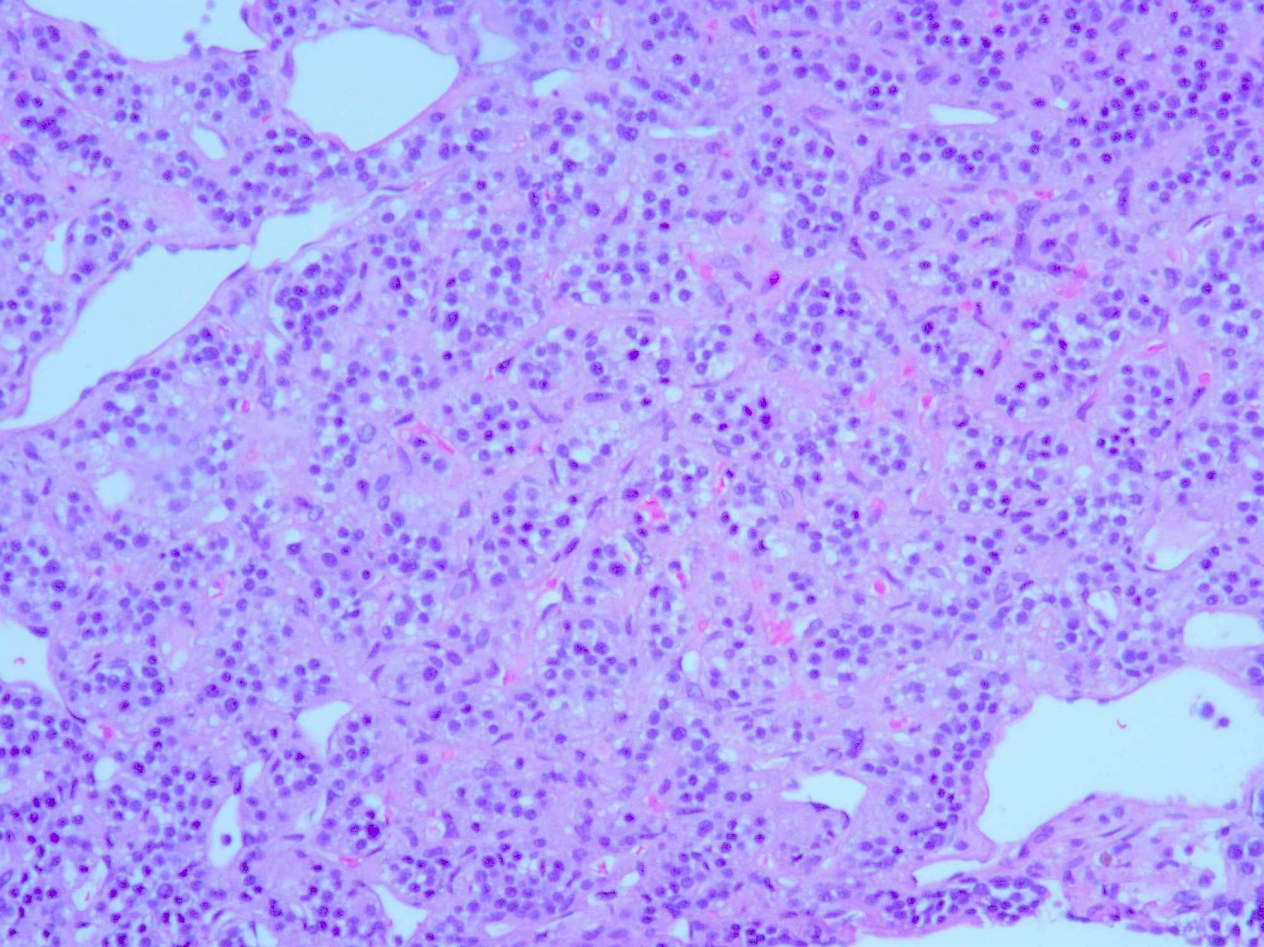
Cells from the biopsy sections of the tumor with hematoxylin and eosin (H&E) stain, 100x amplification, showing nesting of small blue cells with abundant vasculature

**Figure 5:**
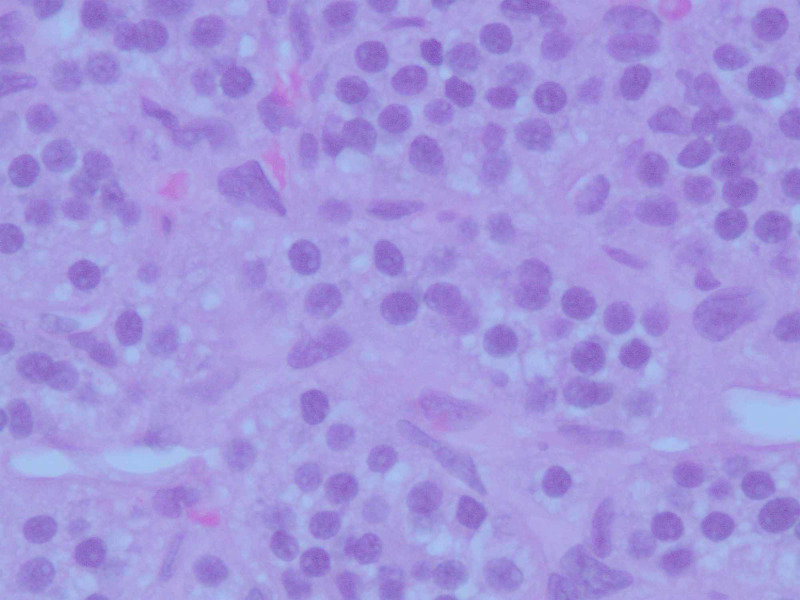
Cells from biopsy sections of the tumor with hematoxylin and eosin (H&E) stain, 400x amplification showing small blue cells with salt, pepper chromatin pattern, and lack of mitosis

**Figure 6:**
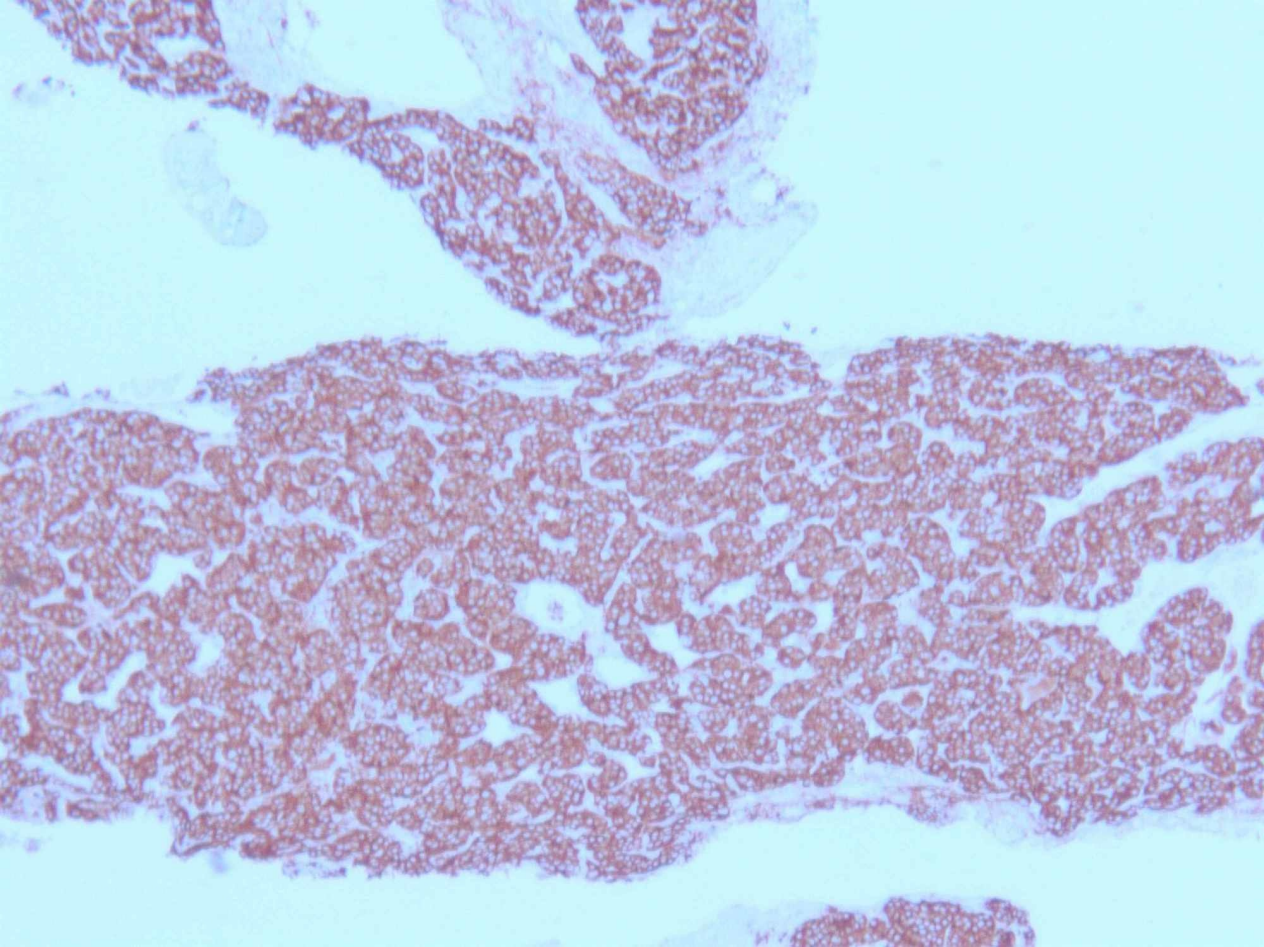
Immuno-histochemistry done on tumor tissue - synaptophysin immunostain is strongly positive for tumor cells

**Figure 7:**
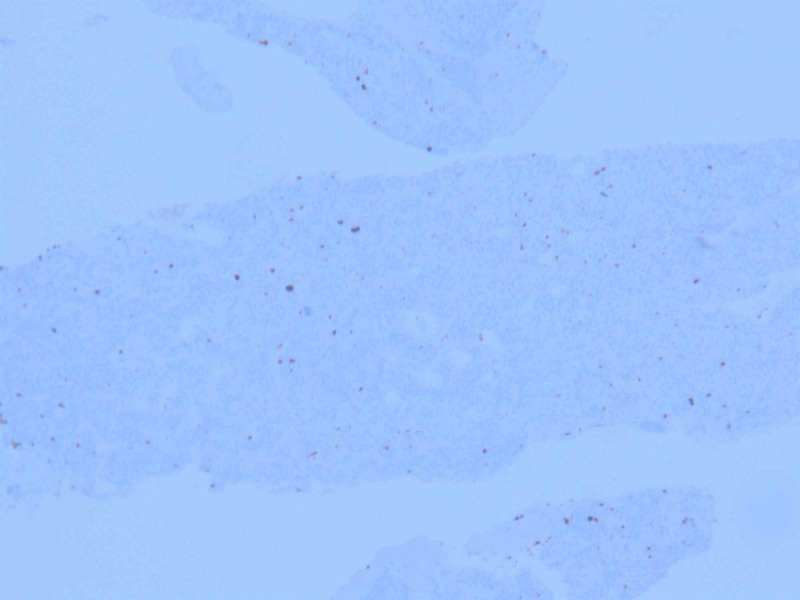
Ki-67 staining on the tumor tissue showing staining of less than 2% of tumor cells

An initial plan for tumor excision via thoracotomy was terminated due to significantly increased risk of bleeding, and other potential complications. A CT-guided biopsy of the mass was performed which showed nesting of small blue cells with salt & pepper chromatin pattern, abundant vasculature, and lack of mitosis (Figures 4-5). Immunostain strongly expressed synaptophysin, chromogranin A, and cluster of differentiation (CD)56 marker (Figure 6). A proliferation marker, Ki-67, was less than 2% and there was no evidence of necrosis within the tumor (Figure 7). The pathology was consistent with grade-I typical thymic NET. The patient underwent embolization of the feeding arteries from the right coronary artery in an effort to reduce the size of the tumor. This was done using Gem foam slurry and subsequently embosphere particles. The tumor size and the mass effect continued to progress while the patient was undergoing sequential embolizations of the feeding arteries. Within a couple of weeks from her presentation, the patient ultimately succumbed to death due to cardiorespiratory arrest secondary to the tumor causing obstructive shock and respiratory failure. 

## Discussion

Our case demonstrates a classic example of a carcinoid mass, often described as “cancer in slow motion” as they can grow slowly for many years without causing symptoms. We encountered an extremely rare thymic NET, thought to be an indolent mediastinal mass at first, which grew insidiously throughout the patient’s mediastinum leading to an acute presentation of severe congestive heart failure (CHF) and eventually leading to death.

Thymic carcinoids affect patients over a wide age range with the majority of patients presenting with symptoms related to mass effect on mediastinal and other thoracic structures. As noted earlier, thymic carcinoids usually occur in the Caucasian male population; however, we report in an African American female, which represents an even more rare instance of the cancer.

Clinical features may include cough, dyspnea, chest pain, and superior vena cava syndrome [7,8]. As high as 50% of thymic NETs have been shown to occur in association with other endocrinopathies such as Cushing syndrome (33%-40%), multiple endocrine neoplasia (MEN) type-1 (19%-25%), and MEN type-2 [9]. At presentation, nearly 20% of patients have metastasis, with extra-thoracic metastasis around 20%-30% [10]. This is likely due to the indolent and slow-growing nature of these tumors as they would not have systemic manifestations and symptoms until later on in the course.

Typical thymic carcinoid is defined by < 2 mitosis/2 mm2 with no necrosis; atypical thymic carcinoid has < 2 mitoses/2 mm2 with necrosis or 2-10 mitoses/2 mm2 with or without necrosis [11]. Markers expressed on thymic NETs on immunohistochemistry are cytokeratin, chromogranin, synaptophysin, neuron specific enolase, Leu-7, and bombesin. Other markers - serotonin, somatostatin, neurotensin, and S-100 protein may also be present [12].

Overall survival was correlated with tumor size, stage, grade, and surgical resection [13]. Current National Comprehensive Cancer Network (NCCN) guidelines recommend surgery when NET is resectable [14]. In an asymptomatic low-grade (typical) thymic NET with locoregional disease, observation or somatostatin analog can be considered. Radiation therapy (RT) +/- concurrent cisplatin-etoposide or carboplatin-etoposide are options for intermediate-grade (atypical) NET with locoregional disease. Everolimus, cisplatin-etoposide, carboplatin-etoposide, or temozolomide +/- capecitabine are preferred treatment options in patients with clinically significant tumor burden and metastatic or progressive disease.

In our case, the patient presented with a large, grossly unresectable low grade mass. Attempts to shrink the mass to reduce the acute effects on the surrounding organs were unsuccessful but if successful, would have been followed by the therapies indicated above.

## Conclusions

Our case illustrates the paradoxical nature of a low-grade thymic NET that insidiously invaded the structures of the mediastinum. Even tumors initially thought to be indolent can become deadly at later stages. Their very nature, slow-growing, would allow them to proliferate slowly for years without causing any major side effects. In our case, this slow growth pattern led to an unfortunate situation 20 years later causing mass effect on the heart and lungs, new-onset heart failure with reduced ejection fraction, and eventual death for our patient. Our case highlights the challenges of harboring a high clinical suspicion even in the face of a seemingly indolent mediastinal mass. Additionally, as our patient was refusing the primary definitive treatment therapies, we address alternate treatment options. Although our modified plan of care was not associated with a positive outcome in our case, the addition of these options may help improve outcomes in the management of such a condition that is rarely seen and may not be properly understood in terms of its appropriate management.
